# Juvenile Dermatomyositis—Clinical Phenotypes

**DOI:** 10.1007/s11926-019-0871-4

**Published:** 2019-12-11

**Authors:** Danyang Li, Sarah L Tansley

**Affiliations:** University of Bath, Claverton Down, Bath, BA2 7AY UK

**Keywords:** Juvenile, Myositis, Autoantibodies, Subgroup, Phenotype

## Abstract

**Purpose of Review:**

Juvenile dermatomyositis is a heterogeneous disease with variable clinical outcomes. Here, we describe the recognised subtypes of idiopathic inflammatory myositis which occur in children, with particular reference to disease-associated autoantibodies.

**Recent Findings:**

Large cohort studies have demonstrated that myositis autoantibodies are common in juvenile dermatomyositis and can be found in the majority of patients. They identify homogenous clinical subgroups and inform prognosis, particularly the risks of developing interstitial lung disease. Descriptions of immune-mediated necrotising myositis in juvenile patients have highlighted a rare but important clinical subset typically associated with severe muscle disease and treatment resistance.

**Summary:**

It is increasingly apparent that autoantibodies can provide detailed information on prognosis and the likely disease associations in those with juvenile dermatomyositis. Further work is needed to establish how this knowledge should influence our approach to treatment.

## **Introduction**

Juvenile-onset myositis is a very rare disease with an incidence of approximately 2–4 per million [1, 2]. The vast majority of affected children have associated cutaneous disease, and, as such, juvenile dermatomyositis (JDM) is often used as an umbrella term for all juvenile-onset myositis. Within the JDM subgroup, there remains significant heterogeneity with variable chronicity, organ involvement and long-term clinical outcome. The Bohan and Peter criteria, proposed in 1975 [3, 4], have been widely used to identify patients; however, they fail to describe the full spectrum of myositis and provide with no definition for clinically amyopathic myositis, immune-mediated necrotising myositis or inclusion body myositis; although the latter is not seen in children. In 2017, the European League Against Rheumatism and American College of Rheumatology published new classification criteria for adult and juvenile idiopathic inflammatory myopathies and their major subgroups. Limitations of the updated criteria include the exclusion of myositis-specific autoantibodies, except anti-Jo-1, and histological features relevant to immune-mediated necrotising myositis (IMNM) [5].

Strategies to better define homogeneous subgroups of patients with JDM are crucial to facilitate diagnosis, inform prognosis and permit good-quality clinical trials of novel and existing therapies. Recent studies have demonstrated that autoantibodies can be identified in 60–95% of patients with JDM and provide a further degree of phenotypic refinement [6••, 7, 8], in addition to histopathological correlations [9]. Here, we discuss the different clinical phenotypes of patients with JDM and suggest autoantibody status as a means of identifying key subgroups, which is summarised in Table 1.

**Table 1.** Clinical subgroups of patients with juvenile-onset myositis. A summary of clinical JDM subgroups with reference to associated autoantibodies and how autoantibody status may influence clinical phenotype and likelihood of response to standard treatment

Description of clinical subgroup	Commonly identified autoantibodies and prevalence in JDM cohorts	Key clinical considerations and influence of autoantibody status on phenotype
Dermatomyositis ( [1–5])		
Skin and muscle disease. Well characterised histological features. Clinical outcome and response to standard treatment variable	Anti-TIFγ 18–32%	Severe cutaneous disease, ulceration, lipodystrophy, muscle disease may be milder. Chronic disease course
	Anti-NXP2 15–23%	Severe muscle disease, gastrointestinal bleeding and calcinosis. Lower remission rates
	Anti-MDA5 7–38%	Arthritis, ulceration, ILD, muscle disease may be milder. Rapidly progressive ILD and increased mortality in East Asian cohorts
	Anti-Mi2 4–10%	Low-incidence organ involvement, severe muscle disease, responds well to standard treatment
Amyopathic dermatomyositis ( [1–5])		
Characteristic dermatomyositis skin manifestations with no muscle involvement. Minimal or progressive muscle involvement is more common than an absence of muscle disease	Anti-MDA5 7–38%	See above
	Anti-TIF1γ 18–32%	See above
	Anti-SAE 1%	Muscle involvement may develop later
Immune-mediated necrotising myositis ( [2, 3, 7])		
Severe muscle disease, histology shows myofibre necrosis with minimal inflammatory cell infiltrate. Note that rash has been reported in some JDM patients but can be atypical.	Anti-SRP 2%	Severe muscle disease, cardiac involvement, treatment resistance
	Anti-HMGCR 1%	Severe muscle disease, treatment resistance
Anti-synthetase syndrome ( [2, 3])		
Myositis, ILD, Raynaud’s phenomenon, arthritis, fever and mechanic’s hands	Anti-Jo-1, anti-PL7, anti-PL12, anti-OJ, anti-KS, anti-EJ, anti-Zo, anti-Ha Collectively < 5%	Different anti-synthetase autoantibodies are associated with muscle-dominant or lung-dominant disease in adult patients. Phenotype data for JDM are limited.
Overlap disease ( [2, 3])		
Patients fulfil classification for myositis and another rheumatic disease. Commonly, systemic sclerosis, inflammatory arthritis and systemic lupus erythematosus.	Anti-PmScl (4–5%) Anti-U1RNP (4–6%) Also reported anti-Ku, anti-U3RNP and anti-Scl70	Caution that due to overlapping clinical features patients may more appropriately fit

ILD, interstitial lung disease

## Classic Dermatomyositis

Anti-Mi2 is generally considered the archetypal dermatomyositis autoantibody. Affected patients typically present with significant skin and muscle involvement and have a low incidence of additional organ involvement. These patients have histologically severe disease on muscle biopsy with high overall severity scores and characteristic histological features [10•]. Interestingly, while anti-Mi2-associated JDM is often severe at presentation, these patients typically respond well to conventional therapy and are considered to have good prognosis [6••].

Most patients with JDM who present with classic dermatomyositis will not have anti-Mi2 disease as this autoantibody is found in just 4–10% of affected patients [6••]. While often presenting similarly with both skin and muscle involvement, patients with anti-NXP2 (15–23% of patients) and anti-TIFγ (18–32% of patients) are more likely to be resistant to conventional therapies [6••, 7, 11].

Patients with anti-NXP2 have more severe muscle disease [6••, 8, 11, 12] in addition to an increased risk of gastrointestinal bleeding, ulcers and dysphagia [7]. Disease outcome also appears to be worse in this group with more persistent disease activity and a worse functional status [7, 11, 12]. A recent study exploring the clinico-pathological subgroups of JDM found that anti-NXP2 occurred more often in a subgroup of patients with severe muscle weakness, gastrointestinal involvement and prominent severe ischaemic features on muscle biopsy [13]. Furthermore, patients with anti-NXP2 required treatment that was more aggressive and had a lower remission rate during the follow-up period [13]. Calcinosis is a recognised complication of dermatomyositis that is more frequently seen in juvenile-onset disease. Anti-NXP2 autoantibodies have been associated with the development of calcinosis in UK patients, but the association bordered on significance when a larger group of patients was analysed, and this has not been replicated in US studies [6••, 7, 14]. Calcinosis is a cause of significant morbidity, and, in addition to autoantibody status, a recent study using the CARRA legacy registry has linked calcinosis to prolonged active disease, severe disease and clinical features, such as lipodystrophy and joint contractures [15]. Whether anti-NXP2 autoantibodies are directly associated with calcinosis or prolonged active disease leading to calcinosis remains to be seen, but there are increasingly strong arguments for managing such patients aggressively.

In contrast, patients with anti-TIF1γ may have milder muscle involvement [6••, 7]. This subgroup has been associated with more severe cutaneous disease, including ulceration, and, in US studies, lipodystrophy, a late complication of JDM and associated with a severe, chronic disease course [7, 8, 16, 17]. In the UK, patients with anti-TIF1γ are more likely to receive treatment with IV cyclophosphamide, typically reserved for patients with severe disease, although the clinical drivers behind treatment choices remain unknown [6••]. Adult myositis patients with anti-TIF1γ are at increased risk of malignancy within three years of diganosis, but this association is not seen in JDM [6••, 18].

Anti-MDA5 autoantibodies have also been described in JDM, and affected patients typically have milder muscle disease, both clinically and histologically [19]. Importantly, this group has an increased likelihood of developing interstitial lung disease in addition to ulceration and arthritis [19–22]. The prevalence of anti-MDA5 is variable and appears to depend on ethnicity and/or place of origin with 7% of UK children having this autoantibody compared to up to 38% in Japanese JDM cohorts [8, 19, 21, 22]. Rapidly progressive interstitial lung disease and a high associated mortality have been reported in juvenile East-Asian cohorts with anti-MDA5 but not in a predominantly Caucasian UK cohort [19, 21, 22]. A recent US study suggested that the co-existance of anti-MDA5 with anti-Ro52 autoantibodies in JDM patients significantly increased the risk of interstitial lung disease which developed in 9% of those with anti-MDA5 and 70% of those with anti-MDA5 and anti-Ro52 [23••]. One patient with both autoantibodies developed rapidly progressive interstitial lung disease [23••]. The identification of anti-MDA5 autoantibodies, particularly in conjunction with anti-Ro52, should prompt careful monitoring for the development of interstital lung disease.

Thus, while anti-Mi2 patients are likely to be best managed by standard conventional therapies, those with anti-NXP2 and anti-TIF1γ may benefit from an alternative, more aggressive treatment approach. Patients with anti-MDA5 autoantibodies should be carefully monitored for the development of interstitial lung disease.

## Amyopathic Myositis

Amyopathic or clinically amyopathic dermatomyositis describes those patients with characteristic dermatomyositis skin manifestations in the absence of muscle involvement, as defined by clinical examination, muscle enzymes and muscle biopsy. Amyopathic myositis is recognised in children, but it is rare, and, more commonly, patients have mild or progressive muscle involvement [24, 25]. The relationship between skin and muscle disease is complex; patients may present with skin disease alone and subsequently develop muscle involvement, a pattern which has been described in adults and children with anti-SAE autoantibodies [6••, 26••]. In contrast, those who initially have muscle dominant disease may later have persistent skin disease despite remission of muscle disease. Residual skin disease in JDM is now recognised as important and is associated with a longer time to remission [27]. Skin changes should be regularly monitored as part of standard care and actively treated, even in the absence of muscle disease activity [27].

Anti-MDA5 autoantibodies are associated with amyopathic myositis in adult and juvenile cohorts [19–22]. Anti-TIF1γ has also been reported in patients with amyopathic and hypomyopathic myositis but to a lesser degree and mainly in adult patients [28, 29]. In a small Japanese cohort of patients with JDM, muscle weakness was absent in 40% of anti-MDA5 patients and 44% of anti-TIF1-γ at presentation; however, all but one patient with anti-MDA5 autoantibodies developed muscle involvement during follow-up [8].

Dermatomyositis skin changes in the absence of or associated with minimal muscle disease should prompt investigation for myositis autoantibodies. The identification of anti-MDA5 autoantibodies can be helpful in identifying those patients at increased risk of interstitial lung disease, and further investigation should be considered. Patients with amyopathic JDM frequently develop muscle involvement at a later stage.

## Immune-Mediated Necrotising Myositis

IMNM is a relatively recently described subtype of myositis characterised by myofibre necrosis, with minimal inflammatory cell infiltrate on muscle biopsy [30]. In those with adult-onset myositis, most patients previously labelled as polymyositis can now be classified as either IMNM or anti-synthetase syndrome. Polymyositis is rare in those with juvenile-onset disease and occurs in less than 8% of patients [31]. Unlike adults, skin disease has been reported in JDM patients who otherwise present as IMNM and should not prevent this diagnosis from being considered. When present associated rashes are often atypical [6••], IMNM typically presents with severe muscle weakness and markedly elevated muscle enzymes. It is associated with autoantibodies directed against signal recognition peptide (SRP) and HMG-CoA reductase (3-hydroxy-3-methyl-glutaryl-coenzyme A reductase (HMGCR), and patients are often resistant to standard therapies.

Anti-SRP autoantibodies are well-described in adult patients, but studies involving children are generally small. Anti-SRP can be found in approximately 2% of JDM patients who can be expected to present with severe weakness, minimal or no skin rash and markedly elevated creatinine kinase [6••, 32]. Cardiac involvement has been reported in adults with anti-SRP and has also been suggested in affected children [7, 33]. Disease outcomes are poor with one study demonstrating that only half of adult patients reached full or near full-strength after 4 years of treatment [34]. One case series of three JDM patients with anti-SRP demonstrated a good response to aggressive treatment with a combination of rituximab, cyclophosphamide and IVIG, which was followed by maintenance methotrexate and intensive daily physical therapy [32].

Two studies have described anti-HMGCR-associated myositis in children. Both reported this to be rare occurring in ~ 1% of all patients [35•, 36•]. Affected patients were similar to those with anti-SRP; they had more severe muscle disease, very high creatinine kinase levels and were treatment resistant [35•, 36•]. Anti-HMGCR is associated with statin use in adult patients, but affected children had not been exposed to statins [32, 33]. Interestingly, while anti-HMGCR-associated myositis is generally associated with a good prognosis, statin-naive adults, who are typically younger, may also be refractory to treatment [37].

Although uncommon, IMNM and the associated autoantibodies, anti-SRP and anti-HMGCR, define an important subgroup of JDM patients who frequently present with severe and treatment-resistant disease. The presentation may be perceived as unusual for JDM, where minimal or absent rash and treatment resistance can lead to diagnostic uncertainty [35•]. The identification of a characteristic autoantibody can be helpful to confirm the diagnosis thus facilitating early aggressive treatment and hopefully preventing long-term disability.

## Anti-Synthetase Syndrome

The anti-synthetase syndrome is characterised by myositis, inflammatory arthritis, interstitial lung disease, Raynaud’s phenomenon, fever and “mechanics hands”; characteristic skin fissuring at the fingertips. Affected patients have autoantibodies directed against tRNA synthetases, a family of cytoplasmic enzymes responsible for catalysing the binding of amino acids to their corresponding tRNA [38]. There are 20 different tRNA synthetases corresponding to the 20 different amino acids, and, thus far, autoantibodies targeting eight have been described in patients with myositis.

Anti-synthetase syndrome is the most common myositis clinical phenotype in adult patients but is rare in children and found in less than 5% of those with JDM [6••, 7]. Affected JDM patients are typically older with a median age at disease onset of approximately 14 years, compared to 6.5 years for JDM overall [6••, 7]. Anti-Jo-1, targeting histidyl tRNA synthetase, is the most common myositis autoantibody in adults and can be identified in 15–30% of patients [33, 38]. The remaining anti-tRNA synthetases, anti-PL7 (threonyl), anti-PL12 (alanyl), anti-OJ (isoleucyl), anti-KS (asparginyl), anti-EJ (glycyl), anti-Zo (phenylalanyl) and anti-Ha (tyrosyl), are rarer, collectively occurring in 10–20% of adult cases [33, 38]. While anti-synthetase syndrome is generally viewed as one syndrome, there are established differences between the clinical associations of the different anti-synthetase autoantibodies [29, 39–44].

The anti-synthetase syndrome is comparatively rare in JDM, and, in the UK cohort, anti-synthetase autoantibodies were identified in just six (1.3%) patients; all patients with an anti-synthetase autoantibody were classified as having JDM overlap and 60% were of Black ethnicity [3]. Three patients had anti-Jo-1, two anti-PL12 and one anti-PL7 autoantibodies [3]. Importantly, half of this group developed interstitial lung disease, and, interestingly, interstitial lung disease occurred exclusively in those with non-Jo-1 anti-synthetase autoantibodies [6••]. In the adult literature, anti-PL7 and anti-PL12, in addition to Black ethnicity, have also been associated with more severe lung involvement [45]. Sabbagh et al. have recently demonstrated that anti-Ro52 in conjunction with an anti-synthetase autoantibody significantly increases the risk of interstitial lung disease in JDM patients [23]. Similarly to our UK cohort, 40% of JDM patients with an anti-synthetase autoantibody developed interstitial lung disease, but this increased to 100% of those with anti-Ro52 and an anti-synthetase autoantibody [23].

Perhaps because of its relative rarity, the anti-synthetase syndrome is less well-recognised, and JDM patients are commonly labelled as myositis overlap disease [18]. The anti-synthetase syndrome is important to identify due to the high incidence of interstitial lung disease [6••, 7, 23].

## Overlap Myositis

Patients can be described as having JDM overlap if they fulfil criteria for both JDM and another rheumatic disease. JDM overlap with systemic sclerosis, inflammatory arthritis and systemic lupus erythematosus have all been widely reported. A potential difficulty with this approach is that rheumatic diseases often have overlapping clinical features. Similarly, the disease phenotype can evolve overtime and affected children can develop features of a related condition as their disease progresses.

Children with anti-synthetase syndrome and anti-MDA5 autoantibodies can be labelled as “myositis overlap” despite the well-characterised myositis disease phenotypes described previously [18, 19]. We suspect that this is because these myositis subtypes are less well-recognised in children due to their comparative rarity, their association with interstitial lung disease and patients frequently do not have muscle dominant disease.

Anti-PmScl autoantibodies are seen in both patients with myositis and systemic sclerosis, and patients frequently have overlapping features of both diseases. Unlike in adult patients, those with JDM and this autoantibody commonly have dermatomyositis-related skin rashes [6••]. Anti-U1RNP is another autoantibody typical of overlap disease that is common in JDM and particularly older children. Anti-U1RNP is also found in patients with lupus and mixed connective tissue disease. Both these autoantibody subgroups are relatively common in JDM and can be found in 5 and 4% of UK children, respectively.

## Conclusion

A number of clinically defined subgroups can be seen in patients with juvenile-onset myositis. These subgroups can inform prognosis, further investigations and treatment approach, but the vast majority of patients fall into the classic dermatomyositis category which remains very heterogeneous. Autoantibodies are identifiable in the majority of JDM patients and enable a further degree of phenotypic refinement beyond clinical subgroups. They are particularly helpful in identifying those patients at high risk of interstitial lung disease and who are more likely to respond poorly to standard treatment. Autoantibodies may also help to provide diagnostic clarity, particularly for disease phenotypes that are rare in children.
